# High Load of Deformed Wing Virus and *Varroa destructor* Infestation Are Related to Weakness of Honey Bee Colonies in Southern Spain

**DOI:** 10.3389/fmicb.2019.01331

**Published:** 2019-06-14

**Authors:** Sandra Barroso-Arévalo, Eduardo Fernández-Carrión, Joaquín Goyache, Fernando Molero, Francisco Puerta, José Manuel Sánchez-Vizcaíno

**Affiliations:** ^1^Animal Health Department, VISAVET Centre, Veterinary School, Complutense University of Madrid, Madrid, Spain; ^2^Apicultural Reference Center in Andalusia (CERA), Andalusia, Spain

**Keywords:** deformed wing virus, *Varroa destructo*r mite, colony strength, honey bee, colony vigor, colony losses

## Abstract

Many factors, including pathogens, contribute to the continuing losses of colonies of the honey bee *Apis mellifera*, which has led to steady population decline. In particular, colony losses have been linked to deformed wing virus (DWV) and the *Varroa destructor* mite. To clarify the potential role of these two pathogens in honey bee colony weakening and loss, we sampled colonies in southern Spain during a 21-month period and analyzed the samples for loads of four viruses and varroa. Loads of DWV and black queen cell virus as well as varroa infestation negatively correlated with colony vigor as measured using the subjective colony strength method. Logistic regression identified varroa and DWV as the main factors involved in colony weakening. Our results confirm that varroa and DWV play a key role in triggering colony weakening in southern Spain and provide evidence that experienced beekeepers’ and technicians’ assessments of colony vigor can accurately estimate colony strength.

## Introduction

In recent years, high annual losses of honey bee colonies and reduced population of native and wild bees have been reported (Genersch, 2010; Ravoet et al., 2013; Goulson et al., 2015; Jacques et al., 2017). These losses have been attributed to colony collapse disorder (vanEngelsdorp et al., 2009) and winter losses of 10–15% worldwide, with reductions in overall colony health, brood density, and total honeybee comb number in some cases (Zee et al., 2014), or even higher ratio of brood to bees (compensatory brood production by diseased colonies; Wegener et al., 2016). Meanwhile, colony losses have reached a level of 30.7% in 2018 in the USA (Bruckner et al., 2018), and mean mortality of European colony losses has remained lower (11.22% in 2014) (Jacques et al., 2016). These losses appear to result from several factors, including pathogens, nutrition, beekeeping management, environment, and pesticides (vanEngelsdorp et al., 2009), as well as colony collapse disorder (CCD) and overwintering (Kielmanowicz et al., 2015).

While a combination of intrinsic and environmental stressors perturbs the equilibrium among honey bee, pathogens, and environment, hive decline appears to depend strongly on pathogens (vanEngelsdorp et al., 2009). Different combinations of pathogens have been identified in dead or depopulated colonies (vanEngelsdorp et al., 2009; Dainat et al., 2012a; Goulson et al., 2015), where pathogens seem to interact through synergy (mutual facilitation) or antagonism (inhibition), or the pathogens may not interact but instead cause additive (cumulative) effects (Wegener et al., 2016). Direct effects of pathogen combinations in the same colony are still poorly understood and may depend on host physiology and apiary environment. For example, varroa seasonality influences load of varroa-related viruses, so the effects of pathogens on honey bee health may vary with the seasons (Tentcheva et al., 2004; Francis et al., 2013).

Widespread all over the world, the *Varroa destructor* mite has adapted to the developmental stages of the honey bee and is a recurrent threat to honey bee populations (Ball, 1993). The mite externally digests and consumes fat body tissue (Ramsey et al., 2019) and feeds on honey bee hemolymph, causing weight loss at individual level. It also damages honey bees indirectly by transmitting several viruses, including deformed wing virus (DWV), Kashmir bee virus (KBV), sacbrood bee virus (SBV), and Israeli acute paralysis virus (IAPV) (Kralj and Fuchs, 2006; Martin et al., 2012). Meta-genomic analysis has suggested that IAPV may cause CCD (Cox-Foster et al., 2007; Maori et al., 2007), but subsequent work in Spanish apiaries has called into question the link between IAPV and colony losses (Blanchard et al., 2008; Garrido et al., 2013; Vicente-Rubiano et al., 2013).

*Varroa destructor* mite can synergize with the DWV picornavirus to trigger colony losses (Nazzi et al., 2012). When varroa infestation levels exceed 2,000–3,000 mites per colony during the autumn season, most over-wintering bees become susceptible to DWV infection, causing colony collapse during the winter. This is because the new brood produced during this period is insufficient to replace the infected over-wintering bees that die (Martin, 2001). Even when larvae affected by DWV and varroa survive, they show altered behavior and learning capacity as well as shorter life span (Iqbal and Mueller, 2007; Benaets et al., 2017; Nazzi and Pennacchio, 2018).

Parasites can reduce colony strength and vigor, rendering the colony more vulnerable to stress-induced disease. Colony strength is typically measured objectively based on certain parameters or subjectively based on visual estimation of a target by one or more observers (Delaplane et al., 2013). These assessments require opening the hive, removing all frames, and allowing substantial time for at least two experts to assess the relevant parameters. Given the difficulty of such assessments in field conditions, beekeepers can instead measure colony vigor, which is defined as strength and health of a living organism (the colony). It can be assessed simply by determination of bee’s activity (visual inspection, emitted sounds; Hatjina et al., 2014).

Load of DWV and levels of varroa can predict colony collapse, and both agents are linked to colony strength (Sumpter and Martin, 2004; Dainat et al., 2012b). Although this fact has been already reported by previous studies (Martin, 2001; Nazzi et al., 2012; Francis et al., 2013; Koziy et al., 2019), few studies have demonstrated this relationship between the mite and the virus with colony weakness in Spain. Most of the works have focused on determining their prevalence without directly linked both pathogens to colony death or weakness. Understanding the dynamics of DWV and varroa and their relationship to colony strength may provide information useful for colony monitoring. This was the objective of the present study, which relied on colony vigor as an alternative way to determine colony strength. Ten *Apis mellifera* colonies in an experimental apiary were sampled during a 21-month period, and samples were evaluated for levels of four viruses and varroa mite. Colony strength and vigor were quantified during sampling. We hypothesized that higher viral load and varroa infestation levels would be associated with poor vigor, low population, lack of brood, and reduced honey and pollen reserves.

## Materials and Methods

### Experimental Setup

The colonies sampled in this study were located in Andalusia in southeast Spain, which contains the second largest number of hives in the country (20% of the colonies in the country) and is one of the country’s most honey-productive regions (around 6 colonies/km^2^). Technicians from the Beekeeping Reference Centre of Andalusia at the University of Córdoba maintained and monthly sampled an experimental *Apis mellifera* apiary from March 2015 to March 2017, except for July and August 2015, when sampling was not possible. In the beginning, the apiary contained 10 colonies, of which 7 collapsed during the study: one colony at May 2015; another colony at June 2015; another colony at August 2015; another colony at April 2016; two colonies at December 2016; and one colony at January 2017. A total of 21 samplings were monthly conducted, generating 142 samples. During sampling, epidemiological data were collected based on colony-level inspection.

### Determination of Colony Strength by a Standardized Method (Subjective Method)

At each sampling, colony strength was measured by two experienced technicians estimating the percentages of cells in each comb that were covered by bees, brood, honey, or pollen, following the subjective method as described (Delaplane et al., 2013). Raw data were converted into colony bee populations as described (Delaplane et al., 2013).

### Determination of Colony Strength by Vigor Measurement

At each sampling, two experienced technicians assessed colony vigor on a scale from 1 (least vigorous) to 10 (most vigorous). A score of 1 was associated with colonies with a small population occupying less than the space between three frames and having low amount of stores, in the form of nectar/honey and pollen. A score of 10 was given to colonies with a large population of bees, occupying more than the space between 6 and 7 frames and having ample stores. Worse health status indicators included obvious presence of clear DWV disease symptoms, such as deformed wings and shortened abdomen and discoloration; diagnosis of any disease potentially associated with immune depletion, such as chalkbrood (Glinski and Buczek, 2003); problems in managing *Varroa destructor* infestation; presence of dead bees around the colony; and a clearly reduction of honey bees.

### Quantification of Bee Viruses

Samples of adult bees were taken from the hive entrance of each colony and frozen at −80°C until analysis. Each sample consisted of a pool of 10 bees, which were homogenized in 5 ml of sterile phosphate-buffered saline (PBS) using a mortar and pestle. This amount of starting material should allow detection of DWV if it is present in more than 25% of bees, with a detection probability of 99% at the colony level (Delaplane et al., 2013). RNA was extracted using the column-based Nucleospin II Virus^®^ kit (Macherey Nagel, Düren, Germany) following the manufacturer’s instructions. Total RNA was suspended in RNase- and DNase-free water and stored at −80°C. This RNA served as template in one-step real-time reverse transcription polymerase chain reaction based on SYBR Green detection for the following viruses: DWV, black queen cell virus (BQCV; Kukielka et al., 2008a), Israeli acute paralysis virus (Maori et al., 2009), and sacbrood bee virus (SBV; Amiri et al., 2015). Viral loads were classified into four categories according to Amiri et al. (2015). DWV primers were previously tested for the detection of a wide variety of DWV.

Loads were quantified in absolute terms. RT-PCR and PCR amplification products of bee pathogens were cloned into a pGemT^®^ vector (Promega, Madison, WI, USA) and transformed into OneShot^®^ TOP10 chemically competent cells (Invitrogen, Carlsbad, California, USA) according to the manufacturers’ instructions. Plasmids were isolated using the QIAprep Spin Miniprep Kit (Qiagen, Venlo, Netherlands) and confirmed as having pathogen-specific inserts using PCR. Plasmid concentrations (ng/μl) were determined using the Nanodrop^®^ apparatus (Thermo Fisher Scientific Waltham, Massachusetts, USA). Standard curves were constructed using triplicate measurements of serial dilutions of known amounts of each plasmid, ranging from 10^10^ to 10^1^ copies/bee (limit of quantitation = 10^1^ copies/bee). Samples for which the dissociation curves showed the same Tm as positive controls but whose Ct values were below the limit of quantitation were considered positive but not quantifiable (Ribière et al., 2002; Gauthier et al., 2007). A virus-free (negative control) and a virus-infected (positive control) sample were included on each assay. Pathogen load was expressed as genome equivalent copies per bee (GEC/bee).

### Quantification of *Varroa destructor* Infestation

*Varroa destructor* load was quantified in all colonies at each monthly sampling throughout the study period, except for July and August 2015, when sampling was not possible. Mite load was quantified using the soapy water method (Dietemann et al., 2013). Briefly, 300 bees were collected from the colony and shaken in a tube containing soapy water and closed with a mesh top. In this procedure, mites detach from honey bee bodies and fall through the mesh. *Varroa destructor* infestation rate was calculated as follows:

$$\begin{array}{l} Varroa\;destructor\;\mathrm{infestation rate}=\\ \;\;\;\;\;\;\;\;\;\;\;\;\;\;\;\;\;\;\;\;\;\;\;\;\;\;\;\;\;\;\;\;\;\;\;\;\;\;\;\;\;\;\;\;\;\;\;\;\;\;\;\;\;\;\;\;\;\;\;\;\;\left(\mathrm{no}.\;\mathrm{of mites}/100\;\mathrm{bees counted}\right). \end{array}$$

After sampling and inspection in September 2015/2016 and March 2016, colonies were treated with oxalic acid against varroa mites.

### *Nosema ceranae* Testing

As *Nosema ceranae* has been related to colony mortality in Spanish apiaries, a subset of the colonies was screened for this pathogen. About 57 of the 142 samples, which corresponded to 7 of the 10 colonies in the period between March 2015 and April 2016, were examined for *Nosema ceranae,* as described in Barroso-Arévalo et al. (2019). *Nosema apis* was not evaluated because of its low prevalence in Spain (Ministerio de agricultura y pesca, a.y.m.a, 2017).

### Statistical Analyses

All data were codified into six categorical variables (Colony ID, sampling, season, death, DWV level, and varroa level) and eight continuous variables (DWV load, BQCV load, *Varroa destructor* infestation rate, bee area, brood area, honey area, pollen area, and colony vigor). Viral loads were log_10_-transformed in order to make patterns more visible.

All statistical analyses were performed using SPSS 22 (IBM, Chicago, USA) and R software for statistical computing, v. 3.1.0 (R Development Core Team, 2014). All statistical tests were set at a significance level of 95%; i.e., *p* < 0.05. First, descriptive analyses was performed. Potential relationships among pathogen loads and between pathogen loads and bee/brood/honey/pollen areas were explored though the Spearman’s correlation coefficient (*ρ*).

Second analysis consisted of survival analysis for investigating the effect of different DWV loads and varroa infestation rates upon the time of the colonies’ collapse. For this analysis, DWV load and varroa infestation rate were classified as categorical variables: high DWV load (RNA equivalents/bee ≥ 10^6^) and low DWV load (10^6^ < RNA equivalents/bee); high varroa infestation rate (varroa infestation rate > 3%) and low varroa infestation rate (varroa infestation rate ≤ 3%) (Bulacio, 2011). As DWV load and varroa infestation rate varied over time, colonies were subclassified into periods according to their health status based on the classification explained above. For instance, if colony 1 showed high DWV load and low varroa infestation rate for 3 months, it was considered as one independent period (1a); if colony 1 showed low DWV load and low varroa infestation rate in the following 4 months, it was considered as another period (1b). Thus, each colony was transformed into different analysis units. This approach allowed us to study the probability of colony collapse in response to variations in DWV and varroa levels. Two Kaplan-Meier curves were created to evaluate the cumulative risk of colony collapse with different DWV load (high/low) and varroa infestation rate (high/low). In addition, Cox regression analysis was performed to detect significant differences in the risk of colony collapse depending on DWV and varroa levels. Colony was considered as a strata variable for the analysis. Cox regression analysis was computed with the survival and survminer packages in R software.

Third analysis focused on exploring the potential influence of DWV load, BQCV load, or varroa infestation rate on colony vigor. A generalized linear mixed model (GLMM) with colony as random effect was conducted between the outcome DWV and BQCV load (quantitative variable: RNA equivalents/bee) and colony vigor. The same analysis was performed with the outcome varroa infestation level (quantitative variable: no. of mites/100 bees counted) and vigor. The random effects examine the difference between colonies with respect to their baseline pathogen level and the changes over time. For this analysis, vigor was classified as a binary variable. As the values for vigor were ranged between 3 and 9, vigor was classified as low if the vigor value was <6 (Vigor = 1) or high if the vigor value was ≥6 (Vigor = 0). GLMM was computed with the lm4 package in R software.

## Results

### Pathogen Loads and *Varroa destructor* Infestation Rate During the Study

**Figure 1:**
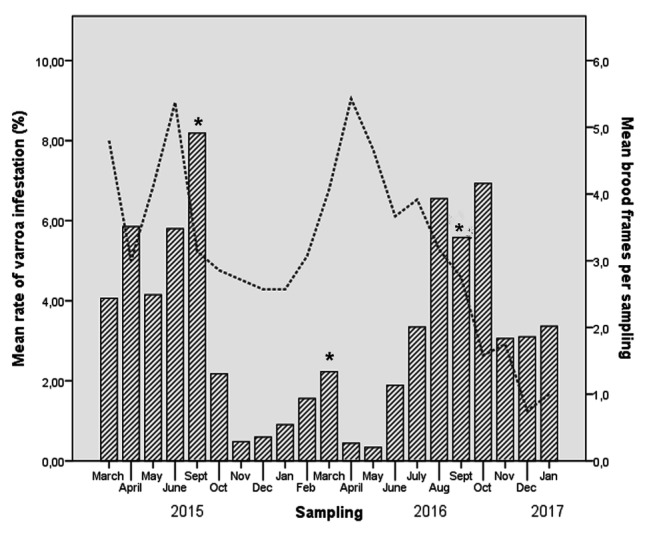
Rates of *Varroa destructor* infestation (per 100 adult bees) and mean of number of brood frames evolution in each colony, based on monthly sampling. Asterisks mark months when anti-*Varroa destructor* treatment was applied.

All samples evaluated for *Nosema ceranae* tested negative. The results showed a high prevalence of DWV and BQCV, while SBV and IAPV were not detected. DWV was present in 139 of 142 samples (97.8%), with an average of 3.39 × 10^5^ GEC/bee (range, 8.32 GEC/bee to 5.25 × 10^9^ GEC/bee). BQCV was present in 113 of 142 samples (79.8%), with an average of 1.58 × 10^3^ GEC/bee (range, 22.1 GEC/μl to 9.83 × 10^9^ GEC/bee). Varroa was detected in at least 80% of samples from all colonies. It was present in 119 of 142 samples (83.8%), achieving its higher levels in September and October. Treatments successfully decreased varroa levels (Figure 1). Comparison between varroa levels and number of brood combs showed that varroa also decreased its levels when the brood dropped (Figure 1).

### Correlations Among *Varroa destructor* Infestation Rate, Pathogen Loads, and Colony Strength

**Figure 2:**
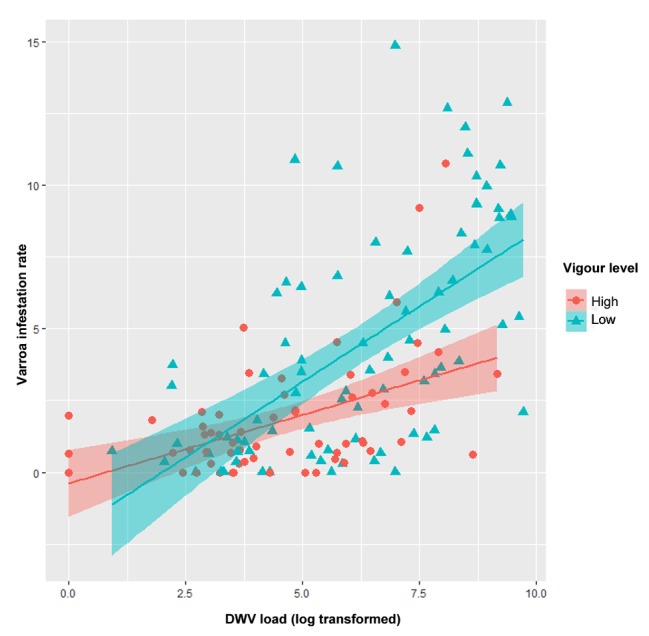
Scatter plot showing correlation of DWV load and varroa infestation rate. Low-vigor colonies are shown in blue; high-vigor colonies, red. Regression lines and confident bands are also shown.

Significant correlations are shown in Table 1. Correlation between *Varroa destructor* infestation rate and DWV load is shown in Figure 2.

**Table 1:** Significant correlations among variables in the study.

Correlated variables	*p*	Spearman’s correlation coefficient (*ρ*)
BQCV, brood area	0.009	−0.305
BQCV, vigor	0.031	−0.252
DWV, brood area	0.003	−0.378
DWV, vigor	<0.001	−0.464
DWV, varroa	<0.001	0.616
Varroa, pollen area	0.011	−0.306
Varroa, vigor	<0.001	−0.474
Bee area, brood area	<0.001	0.610
Bee area, honey area	<0.001	0.686
Bee area, pollen area	<0.001	0.420
Bee area, vigor	<0.001	0.810
Brood area, honey area	0.038	0.210
Brood area, pollen area	0.002	0.317
Brood area, vigor	<0.001	0.747
Honey area, pollen area	<0.001	0.506
Honey area, vigor	<0.001	0.494
Pollen area, vigor	<0.001	0.390

### Survival Analysis

**Figure 3:**
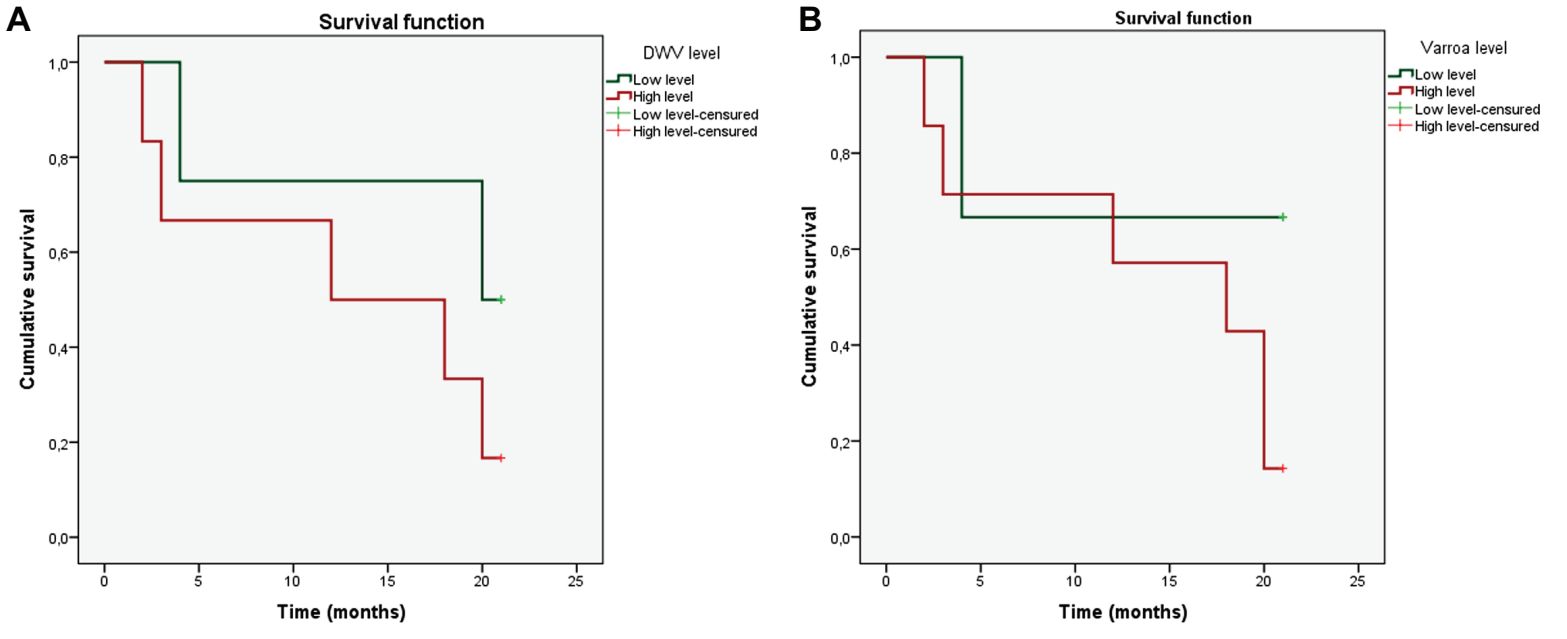
Overall survival according to different DWV **(A)** and *Varroa destructor* **(B)** levels. Green color represents low levels of pathogen; red color represents high levels of pathogens.

Kaplan-Meier plots are showed in Figure 3. Cox regression model showed that DWV load (*p* = 0.0166) and varroa infestation rate (*p* = 0.0481) were independent influencing factors affecting colony collapse. High DWV load was associated with a significantly increased mortality risk (hazard risk, 13.48; 95% confident interval) as well as high varroa infestation rate (HR, 63.95; 95% CI). Most of the colonies collapsed after 4 months with high DWV load and high varroa infestation rate.

### Generalized Mixed Model to Examine Potential Relationships of Virus Loads or Varroa Infestation Rate With Colony Vigor

Colony vigor was significantly associated with DWV load and *Varroa* infestation rate, but not with BQCV load. Varroa infestation rate was significantly higher in those colonies with reduced vigor (*t* = 1.303, *p* = 0.007) as well as DWV load (*t* = 1.167, *p* = 0.017).

### Presence of Disease Symptoms

Some colonies showed symptoms of DWV (such as deformed wings and shortened abdomen and discoloration) and ascospherosis and/or excessive presence of varroa in the phoretic stage, especially in December, January, and February. However, the presence of DWV symptoms or ascospherosis did not correlate significantly with viral load or varroa infestation rate.

## Discussion

In the present study, we analyzed relationships among the loads of four viruses and *Varroa destructor* mite, colony vigor, colony strength (in terms of bee/brood/honey/pollen area), and presence of symptoms in an experimental apiary during a 21-month period from March 2015 to January 2017.

RT-qPCR analysis revealed a high prevalence of DWV and BQCV, while IAPV and SBV were not detected. The total absence of IAPV during the entire study suggests that the virus is not abundant in Spain, at least not in the province of Córdoba. The fact that we can exclude IAPV as a cause of the total loss and depopulation of several colonies during the study is reminiscent of our 2013 observation, also in Andalusia, that IAPV was not involved with colony collapse in that study (Vicente-Rubiano et al., 2013). Our results suggest that the association between IAPV and CCD observed in the USA (Cox-Foster et al., 2007; Maori et al., 2007) may not apply to southern Spain.

BQCV was quite prevalent in our study. This result agrees with those obtained previously in Andalusia and other parts of Spain (Kukielka et al., 2008b; Cepero et al., 2014). DWV was found in the majority of samples, which is higher than the prevalence previously reported in northern and central Spain (Gisder et al., 2009), but agrees with the results from the annual report about honey bee losses performed in the country, where DWV was found in a 99% of the apiaries analyzed (Ministerio de agricultura y pesca, a.y.m.a, 2017). The low prevalence in that previous work may be related to the low presence of *V. destructor* in those apiaries. In our study, varroa was found in 83.8% of samples. This apparent correlation between DWV load and varroa infestation rate agrees with previous studies in Spain (Kukielka et al., 2008a; Vicente-Rubiano, 2015). Varroa have to injure honey bees directly by consuming body tissue (Ramsey et al., 2019) and feeding on their hemolymph, and it can impact bees by transmitting viruses and activating viral replication (Yang and Cox-Foster, 2005; Kralj and Fuchs, 2006). The combination of high DWV load in bees during the summer-autumn season may reflect varroa dynamics and reproduction inside brood cells. As the number of brood increases from spring to summer, mite reproduction increases, peaking in late summer (Evans and Cook, 2018). These considerations suggest that DWV and varroa may act together to affect colony health (Di Prisco et al., 2016) and that these negative effects are likely to be greater when brood is growing, since varroa is reproducing and thereby helping DWV to replicate (Wegener et al., 2016). Virus-parasite-host immunity synergism can profoundly alter colony response to pathogens (Nazzi et al., 2012; Di Prisco et al., 2016). Cox regression analysis and Kaplan-Meier curves showed that after short periods (between 2 and 4 months) with high DWV load and high varroa infestation rate, colonies were more likely to collapse. This fact emphasize the need to monitor DWV and varroa levels in apiaries, as a preventive measure.

We found that colonies that died during the study period showed significantly lower bee and brood areas and significantly lower vigor than colonies that survived. Vigor negatively correlated with varroa infestation rate and DWV load, as well as DWV and brood area, suggesting that the mite-virus complex may predispose the colony to depopulation and weakness. However, these results need to be interpreted with caution, since correlation coefficients were low for those variables (Table 1) and, therefore, their value may be limited. Generally, colonies with high varroa infestation and DWV infection are more likely to experience stress and are therefore more vulnerable to depopulation and less vigor. BQCV also negatively correlated with colony vigor. Since BQCV infection is often asymptomatic and therefore covert (Tentcheva et al., 2004), this correlation may reflect immunosuppression caused by other factors, such as varroa infestation or DWV infection. This immunosuppression can facilitate BQCV replication. In this way, opportunistic viruses like BQCV may actively replicate in bee brood that is infected with DWV and infested with varroa, leading to strong BQCV infection in adult bees (Chen and Siede, 2007; Gauthier et al., 2007).

We ran a generalized linear mixed model to determine whether viral load and varroa infestation rate could explain the observed decreases in colony vigor. The model suggested that, indeed, high varroa infestation rate and high DWV load were related to reduced colony vigor and therefore unstable health status. These results confirm several previous studies documenting a relationship between DWV and the mite (Bowen-Walker et al., 1999; Martin, 2001; Dainat et al., 2012a; Locke et al., 2014). This interaction appears to involve a synergy mediated by host immunity (Nazzi et al., 2012), which is consistent with our observation of overt symptomatology of varroosis and wing deformities in the joint presence of DWV and varroa. Therefore, exhaustive measures to control varroa should be implemented in order to decrease DWV infection.

Seasonal trends were detected for varroa infestation: the mite reached its major infestation level in the spring-summer season, suggesting that the mite contributes to pathogen presence and replication during this season (Yang and Cox-Foster, 2005). Bee/brood/pollen/honey areas were also higher in this season, which may help the mite expand rapidly to the colonies (Wegener et al., 2016; Nurnberger et al., 2019).

Pollen area negatively correlated with varroa infestation rate. A decline in pollen harvest has been associated with a direct reduction in brood production, which may have a negative impact on the adult bee population size, and lack of honey reserves before overwintering. Consequently, colony health can be affected, making the colony more vulnerable to diseases like varroosis (Requier et al., 2017). Individual bees infested with varroa during their development usually survive to emergence but may show signs of physical or physiological damage as adults (Barron, 2015). The feeding and reproduction of varroa mites on developing larvae and pupae of worker brood may have a major effect on adult bee activity (Dainat et al., 2012b; Requier et al., 2017). The mite can trigger wing deformities as well as behavioral alterations that reduce foraging activity, reducing pollen collection. A recent study has suggested that pollen supplementation may provide the survival of honey bee colonies infested by the parasite (Annoscia et al., 2017). Therefore, practical use of pollen for the prevention of varroa infestation should be further explored.

We found that assessment of colony strength measured subjectively (Delaplane et al., 2013) gave similar results as assessment of colony vigor. In addition, vigor correlated positively with bees/brood/pollen/honey area, suggesting that vigor estimation by an experienced technician may be a more straightforward and less invasive method for estimating colony strength.

## Conclusions

We showed that DWV, BQCV, and varroa were highly prevalent at all samplings. DWV load correlated with varroa infestation rate, and high load of both agents was related to lower colony vigor. While our results suggest that the mite-virus interaction contributes to colony weakening, more studies are needed to examine whether interaction with other pathogens also plays a role.

## Data Availability

The datasets used and/or analyzed during the current study are available from the corresponding author on reasonable request.

## Author Contributions

SB-A, JG, and JS-V conceived this research and designed the experiments. FP and EC participated in the design and interpretation of the data. SB-A and FR performed the experiments and analysis. SB-A and EC wrote the paper and participated in the revisions of it. All authors read and approved the final manuscript.

### Conflict of Interest Statement

The authors declare that the research was conducted in the absence of any commercial or financial relationships that could be construed as a potential conflict of interest.
